# Ser-Tyr and Asn-Ala, vasorelaxing dipeptides found by comprehensive screening, reduce blood pressure via different age-dependent mechanisms

**DOI:** 10.18632/aging.102400

**Published:** 2019-11-04

**Authors:** Daiki Koyama, Xinghui Sun, Masaki Sasai, Shigenobu Matsumura, Kazuo Inoue, Kousaku Ohinata

**Affiliations:** 1 Division of Food Science and Biotechnology, Graduate School of Agriculture, Kyoto University, Gokasho, Uji, Kyoto 611-0011, Japan

**Keywords:** aging, dipeptide library, structure-activity relationship, vasorelaxation, nitric oxide

## Abstract

To understand the changes in physiological responses due to aging, a number of bioactive probes based on different signal transduction pathways are necessary. In this study, we comprehensively and systematically investigated changes in blood vessel function with age using a 336-dipeptide library.

In the early stage of hypertension, the most potent vasorelaxant dipeptide was Ser-Tyr (SY) in the mesenteric artery isolated from spontaneously hypertensive rats (SHR). SY-induced vasorelaxation and anti-hypertensive effects were blocked by L-NAME, an inhibitor of nitric oxide synthase (NOS), suggesting that SY activates the NO system. On the other hand, the patterns of dipeptides with vasorelaxation activity in early and advanced stages of hypertension were different. In the advanced stage, the most potent vasorelaxing dipeptide was Asn-Ala (NA). Orally administered NA (1.5 mg/kg) reduced the blood pressure in the advanced stage, at which drugs were sometimes less effective, and the anti-hypertensive effects lasted for 6 hr. The NA-induced vasorelaxation and anti-hypertensive activity was blocked by lorglumide, an antagonist of the cholecystokinin CCK_1_ receptor, suggesting that NA activated the CCK system.

Taken together, in the early and advanced stages of hypertension, SY and NA exhibited vasorelaxing and anti-hypertensive effects via the NO and CCK systems, respectively.

## INTRODUCTION

Aging attenuates many physiological functions and is a major risk factor for many diseases. However, the mechanism of aging-induced physiological alteration has not yet been investigated in details. To increase the healthy life expectancy, the mechanisms of aging must be clarified.

The Canadian physician William Osler said that “A man is as old as his arteries”. Aging is closely associated with functional changes in arteries, and the prevalence of hypertension increases with age [1]. The purpose of this study was to understand the changes in arterial function with age by comparing the vasorelaxation activity in SHR with early (15–18 weeks) and advanced hypertension (over 27 weeks old).

To understand changes in physiological responses caused by aging, a number of bioactive probes based on different signal transduction pathways are necessary. It is known that a number of peptides produced by the enzymatic digestion of food proteins exert physiological functions, including anti-hypertensive, appetite-regulatory, anxiolytic, and antidepressant-like effects [2–5]. Dipeptides, minimal peptides with two amino acids linked, also exhibit physiological functions. Indeed, we found that Arg-Phe (RF) and Phe-Trp (FW) relaxed the mesenteric artery and reduced blood pressure [6, 7]. However, the vasorelaxation activity of dipeptides has not been comprehensively measured.

Thus, we used a commercially available library composed of 336 dipeptides for the comprehensive measurement of vasorelaxation activity. To effectively investigate the vasorelaxation activity of dipeptides, we prepared dipeptide mixtures consisting of the specific N-terminal or C-terminal residues. In this study, the dipeptide library improved our understanding of functional changes in arteries with age.

## RESULTS

### Comparison of vasorelaxation activity of dipeptide mixtures in SHR with early or advanced hypertension

**Figure 1:**
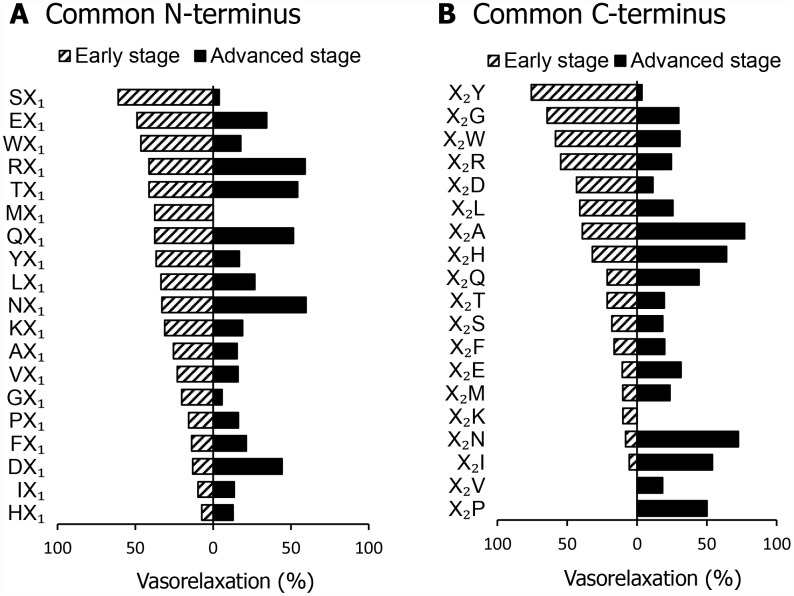
**Vasorelaxing activity of dipeptide mixtures in a mesenteric artery isolated from spontaneously hypertensive rats (SHR) in the early or advanced stage.** Common N-terminal (**A**) or C-terminal (**B**) dipeptides (1 μM) were mixed and vasorelaxing activities were measured. Values are mean (n = 2-3).

We measured the vasorelaxation activity of 38 dipeptide mixtures, which consisted of the specific N-terminal or C-terminal residues, using the mesenteric artery of SHR with early or advanced hypertension. As shown in Figure 1A and 1B, the vasorelaxation activity of the dipeptide mixtures markedly differed between SHR with early and advanced hypertension. In SHR with early hypertension, the dipeptide mixture that consisted of the C-terminal tyrosine residue (X_2_Y) exhibited the most potent vasorelaxation effects. In contrast, in SHR with advanced hypertension, the dipeptide mixtures that consisted of the N-terminal asparagine residue (NX_1_) or C-terminal alanine residue (X_2_A) exhibited the most potent vasorelaxation effects.

### Discovery of potent vasorelaxant dipeptides, SY and NA

**Figure 2:**
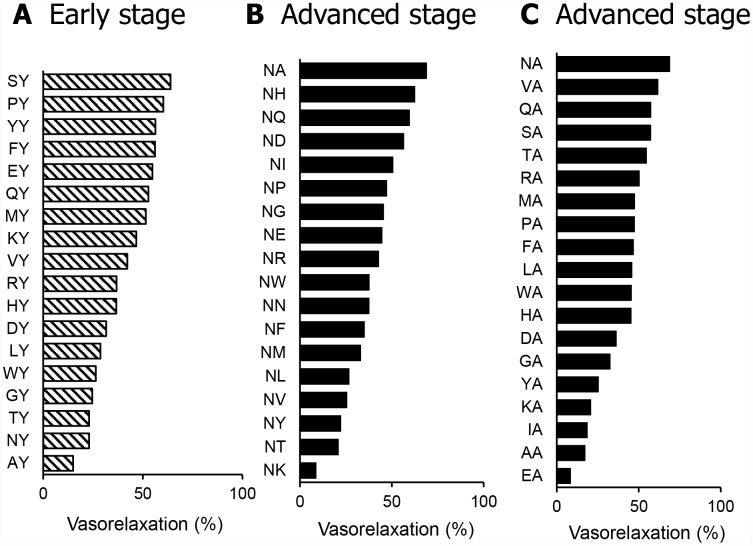
Vasorelaxing activity of dipeptides in SHR mesenteric arteries in the early (**A**) or advanced stage (**B** and **C**). Dipeptides from dipeptide mixtures with the most potent vasorelaxing activity were used at a dose of 1 μM. Values are means (n = 2-4).

In SHR with early hypertension, we measured the vasorelaxation activity of each dipeptide of X_2_Y. As shown in Figure 2A, SY exhibited the most potent vasorelaxation activity. In SHR with advanced hypertension, we measured the vasorelaxation activity of each dipeptide of NX_1_ and X_2_A, and NA exhibited the most potent vasorelaxation activity, as shown in Figure 2B and 2C.

### Aging affects SY- and NA-induced vasorelaxation activity

**Figure 3:**
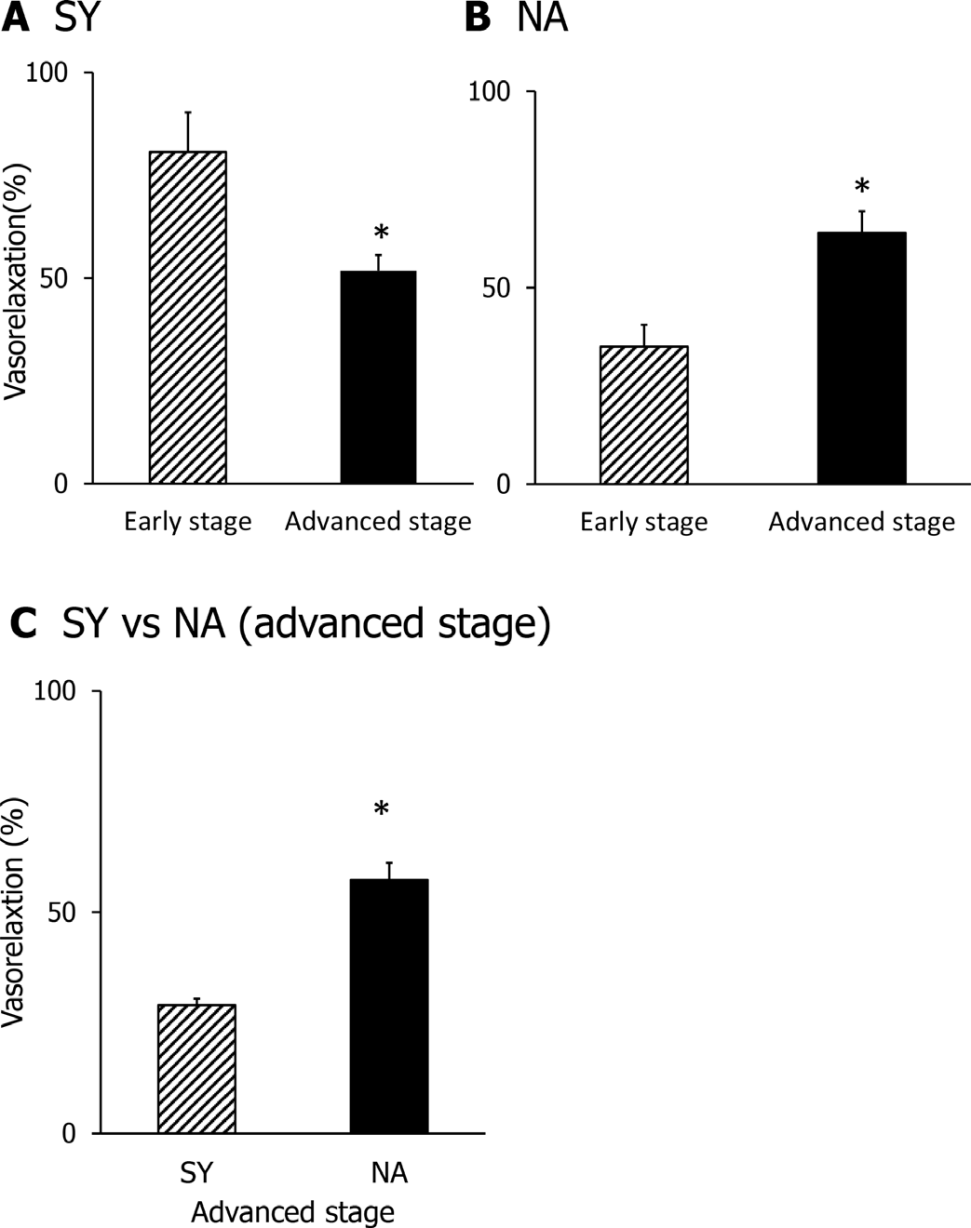
Comparison of the vasorelaxing activity of SY (**A**) and NA (**B**) in the early stage or advanced stage. Comparison of SY and NA in the advanced stage (**C**). Dipeptide was applied at a dose of 1 μM. Values are expressed as the means ± SEM (**A**, n=3; **B**, n =4-5; **C**, n = 3). *P < 0.05, compared with the early stage group (**A**–**B**). *P < 0.05, compared with SY group (**C**).

Next, we investigated whether SY- and NA-induced vasorelaxation activity was affected by aging. SY-induced vasorelaxation activity was significantly attenuated in SHR with advanced hypertension, as shown in Figure 3A. In contrast, NA-induced vasorelaxation activity was higher in SHR with advanced hypertension (Figure 3B). Furthermore, we directly compared the vasorelaxing activity of NA and SY (Figure 3C). NA-induced vasorelaxation activity was significantly greater than that induced by SY in SHR with advanced hypertension. These results support that aging affects the function of arteries.

### SY exhibits vasorelaxation and hypotensive effects via the NO pathway

**Figure 4:**
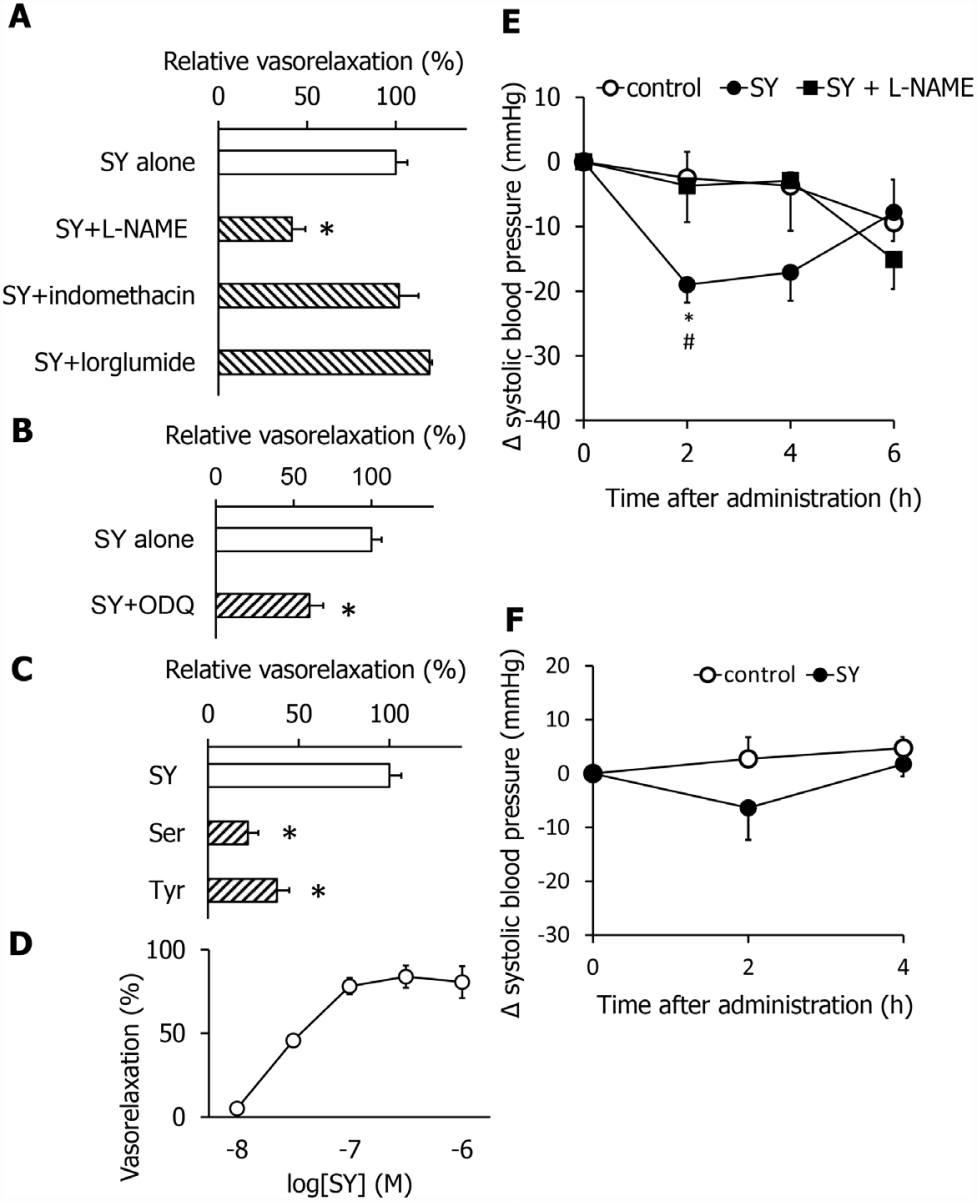
**SY exhibits vasorelaxing and anti-hypertensive effects by activating the NO system in SHR in the early stage.** Effects of different blockers of vasorelaxing activity of SY (**A** and **B**). Comparison of vasorelaxing effects of SY, serine and tyrosine (**C**). The dose dependency of the vasorelaxing activity of SY (**D**). SY-induced anti-hypertensive effects were blocked by L-NAME, an NO synthase inhibitor in the early stage (**E**). Effects of SY on blood pressure in the advanced stage (**F**). Values are expressed as the means ± SEM (**A**, n = 4-5; **B**, n = 4-5; **C**, n = 3; **D**, n = 2-3; **E**, n = 14-16; **F**, n = 7-8). *P < 0.05 compared with the SY group (**A**–**C**). *P < 0.05 compared with the control group (**E**). #P< 0.05 compared with the SY + L-NAME group.

To explore the mechanism of the vasorelaxation effects of SY, we used three vasorelaxation inhibitors, lorglumide, indomethacin, and L-NAME, in combination with SY. The SY-induced vasorelaxation activity was not blocked by lorglumide, an antagonist of the cholecystokinin CCK_1_ receptor, or indomethacin, a cyclooxygenase inhibitor. In contrast, the activity was inhibited by L-NAME, a NO synthase inhibitor (Figure 4A). This suggests that SY exhibits vasorelaxation activity via the NO pathway, which is independent of known CCK and prostaglandin systems. It is known that NO stimulates guanylate cyclase in the vascular smooth muscle to produce cGMP, which induces vasorelaxing effects. ODQ, an inhibitor of guanylate cyclase, also blocked the SY-induced vasorelaxing effects (Figure 4B). Thus, we demonstrated that SY stimulates the NO/cGMP pathway. Next, we investigated the dose dependency. As shown in Figure 4D, SY dose-dependently relaxed the mesenteric artery. To further investigate whether SY exhibits hypotensive effects, we measured systolic blood pressure (SBP) using the tail-cuff method. Orally administered SY (5 mg/kg) significantly reduced the SBP in young SHR (Figure 4E). The amino acids composing the dipeptide SY did not exhibit vasorelaxing effects (Figure 4C), suggesting that the partially absorbed dipeptide acts on the vascular system. In addition, the hypotensive effects of SY were inhibited by pretreatment with L-NAME (20 mg/kg, i.p.) (Figure 4E). Therefore, SY-induced hypotensive effects and vasorelaxation effects are mediated by the NO pathway. The antihypertensive effects of oral SY in SHR with advanced hypertension were weaker than those in SHR with early hypertension (Figure 4F).

### NA exhibits vasorelaxation activity and hypotension in a CCK-dependent manner

**Figure 5:**
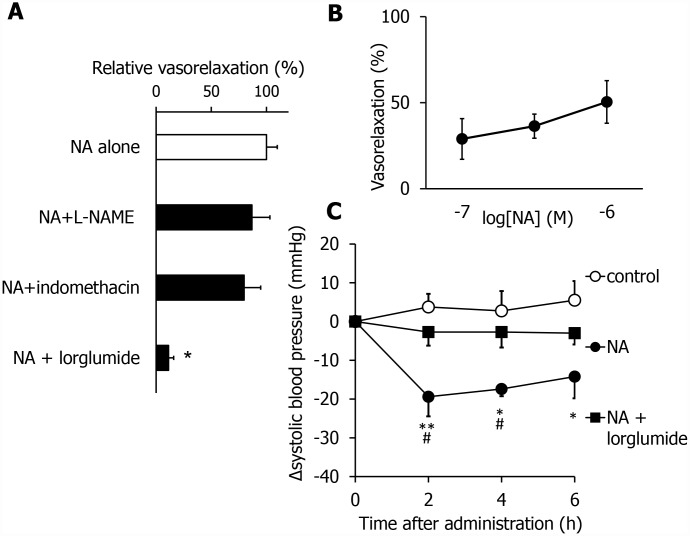
**NA exhibits vasorelaxing and anti-hypertensive effects by activating the CCK system in SHR in the advanced stage.** Effects of different inhibitors of vasorelaxing activity of NA (**A**). The dose dependency of the vasorelaxing activity of NA (**B**). NA-induced anti-hypertensive effects were blocked by lorglumide, an antagonist for the CCK receptor (**C**). Values are expressed as the means ± SEM (**A**, n = 3-4; **B**, n = 5-8; **C**, n=4-7). *P < 0.05, compared with the NA group (**A**). *P < 0.05, **P < 0.01, compared with the control group. #P< 0.05 compared with the NA + lorglumide group (**C**).

To elucidate the mechanism underlying the vasorelaxation effects of NA, we again used three vasorelaxation inhibitors, lorglumide, indomethacin, and L-NAME, in combination with NA. The NA-induced vasorelaxation activity was blocked by neither L-NAME nor indomethacin (Figure 5A). In contrast, the activity was inhibited by lorglumide. These results suggest that NA exhibits vasorelaxation activity in a CCK-dependent manner**,** which is independent of known NO and prostaglandin systems. Next, we investigated its dose dependency. NA dose-dependently relaxed the mesenteric artery, as shown in Figure 5B. To further investigate whether NA exhibits hypotensive effects, we measured the SBP using the tail-cuff method. Orally administered NA (1.5 mg/kg) significantly reduced the SBP in SHR with advanced hypertension (Figure 5C). The hypotensive effects lasted for 6 hrs. In addition, the hypotensive effects of NA were inhibited by pretreatment with lorglumide (Figure 5C). These results suggest that NA has potent and long-lasting hypotensive activity in a CCK-dependent manner.

## DISCUSSION

In the present study, we revealed functional vascular alterations due to aging using a dipeptide library. The profile of vasorelaxation activity of dipeptides was markedly different between SHR with early and advanced hypertension. We also found that SY exhibited the most potent vasorelaxation activity and orally administered SY reduced the blood pressure in SHR with early hypertension, which was mediated through the NO pathway. In contrast, in SHR with advanced hypertension, NA exhibited the most potent vasorelaxation activity, and orally administered NA had long-lasting hypotensive activity in a CCK-dependent manner.

We found that SY strongly relaxed the mesenteric artery from SHR with early hypertension. However, the SY-induced vasorelaxation activity was attenuated in SHR with advanced hypertension. This may be attributed to endothelial dysfunction. Endothelial dysfunction occurs with age and the development of hypertension. Vascular endothelial cells also play important roles in vascular regulation by producing vasorelaxation factors, such as NO, prostaglandin I_2_ (PGI_2_), and endothelium-derived hyperpolarizing factor (EDHF), and vasoconstriction factors, such as endothelin-1 [8]. NO is known to be one of the most important vasorelaxation factors, and the key mechanism of age-related endothelial dysfunction was reported to be reduced responsiveness to NO [9]. Several studies have demonstrated that endothelium-dependent vasorelaxation is attenuated with age [10, 11]. In endothelial cells, NO is synthesized from arginine by endothelial nitric oxide synthase (eNOS). Though whether aging affects eNOS expression is controversial, several studies support that the activity of eNOS decreases with age [12–15]. SY exhibits vasorelaxing and antihypertensive activities via the NO pathway. Thus, in SHR with advanced hypertension, SY-induced vasorelaxing and antihypertensive activities may have been attenuated because of the reduction of NO responsiveness with age.

In contrast to SY, the NA-induced vasorelaxation activity was augmented in SHR with advanced hypertension. This may involve the mechanism of NA-induced vasorelaxation. The vasorelaxation activity of NA was not blocked by L-NAME, which suggests that NA-induced vasorelaxation activity is independent of NO. On the other hand, NA relaxed the mesenteric arteries in a CCK-dependent manner. It was previously reported that CCK exhibits vasorelaxation and antihypertensive effects via the CCK_1_ receptor [16]. We previously reported that a number of CCK-dependent vasorelaxing peptides reduced the blood pressure in SHR [6, 7, 17]. In SHR with advanced hypertension, NA exhibited potent vasorelaxation activity in a CCK-dependent manner, suggesting that the CCK system is important for blood vessels in SHR after the development of hypertension. However, the effects of aging on the CCK system remain unknown, and further studies including molecular markers are needed.

In this study, we used a dipeptide library consisting of 336 commercially available dipeptides. Dipeptides have many physiological functions. Comprehensive measurement of physiological functions by dipeptides provides two important benefits: First, comprehensive measurement of dipeptides revealed functional changes with age. In this study, we found that the profile of vasorelaxation activity of dipeptides was markedly different between SHR with early and advanced hypertension. Moreover, by investigating the mechanisms of vasorelaxant dipeptides, SY and NA, the contribution of NO and CCK systems to vasorelaxation changes with age. Our study demonstrated the effectiveness a “dipeptide library” as a tool to capture functional changes in arteries with age.

Second, information about peptide sequences is helpful to design novel bioactive peptides. We previously found that the dipeptides RF and FW, although not selected in this study, exhibit vasorelaxation and antihypertensive effects. We further identified several potent antihypertensive peptides, such as IHRF from rice glutelin and FWGK from bovine albumin, based on the structure-activity relationship between RF and FW [7, 17]. These longer peptides, although more poorly absorbed after oral administration than dipeptides, exhibited more potent vasorelaxation and antihypertensive effects than dipeptides. Therefore, our results will be useful for the development of novel potent antihypertensive peptides.

In conclusion, we elucidated functional changes in arteries due to aging using a dipeptide library. We also found that SY and NA exhibited the most potent vasorelaxation activity, respectively in SHR with early and advanced hypertension. Orally administered SY and NA reduced the blood pressure. SY-induced vasorelaxation and antihypertensive effects were mediated through the NO production, and NA exhibited vasorelaxation and antihypertensive effects in a CCK-dependent manner. Our results confirmed that a dipeptide library is useful for capturing functional changes with age. To our knowledge, this is the first report of the effects of aging on the cardiovascular CCK system.

## MATERIALS AND METHODS

### Reagents

The dipeptide library was purchased from Anaspec (California, USA). Ser-Tyr (SY) was purchased from BACHEM AG (Bubendorf, Switzerland). Asn-Ala was purchased from RS synthesis (Louisville, KY, USA). Indometacin, a cyclooxygenase (COX) inhibitor, was purchased from Wako Pure Chemical Industries, Ltd. (Osaka, Japan). NG-nitro-L-arginine methyl ester (L-NAME), a nitric oxide (NO) synthase inhibitor, was obtained from Nacalai Tesque, Inc. (Kyoto, Japan). Lorglumide, an antagonist of the cholecystokinin1 CCK_1_ receptor, was from Sigma-Aldrich Co. (St. Louis, MO, USA).

### Dipeptide mixture

We prepared 19 mixtures consisting of dipeptides that had common N-terminal residues. For example, we termed the mixtures prepared from 18 peptides with the same N-terminal alanine residue A as AX_1_ (X_1_ are arbitrary amino acids). We also prepared 19 mixtures consisting of dipeptides that had the C-terminal residue in common.

### Animals

Male SHR/Izm (12–15 weeks old) were purchased from SLC (Shizuoka, Japan). Animals were kept in a temperature-controlled room (23 °C) on a daily 12 hr-light: 12 hr-dark cycle. Rats were fed SP pellets (Funabashi Farm, Chiba, Japan) with free access to water. All studies were performed in accordance with the guidelines for the care and use of laboratory animals of Kyoto University.

### Vasorelaxing assay

The vascular relaxation response was measured as previously described. The small mesenteric artery, 150-200 μm in diameter, isolated from SHR (early stage of hypertension: 15-18 weeks old, advanced stage of hypertension: over 27 weeks old) was cut into helical strips. The artery was suspended in a bathing medium (Krebs-Henseleit solution containing 120 mM NaCl, 4.7 mM KCl, 2.5 mM CaCl_2_, 1.2 mM MgSO_4_, 1.2 mM KH_2_PO_4_, 25 mM NaHCO_3_, and 10 mM glucose) maintained at 37 °C, and bubbled with 95% O_2_ and 5% CO_2_. The artery was constricted with phenylephrine in advance. Vasorelaxation activity was measured in the presence or absence of inhibitors and antagonists, which were applied 5 min before the application of phenylephrine. To determine the complete relaxation point, 100 μM papaverine was added at the end of each experiment.

### Blood pressure measurement

Systolic blood pressure (SBP) after oral administration of SY or NA to conscious SHR (less than 25 weeks old or over 30 weeks old) was measured by the tail-cuff method using a MK-2000 (Muromachi Kikai Ltd, Tokyo, Japan). Before experiments, the animals were trained to undergo measurements by the tail-cuff method for 3 weeks. SY and NA dissolved in physiological saline were used. L-NAME and lorglumide dissolved in physiological saline were administered intraperitoneally (i.p.) just before measuring SBP. SBP was measured just before oral administration, and at 2, 4, and 6 h after oral administration.

### Statistical analysis

Values are expressed as the means ± SEM. Analysis of variance (ANOVA) followed by Tukey-Kramer's test or Dunnett’s multiple comparison, or the Student's t-test were used to assess differences among 3 or more, or two groups, respectively. P < 0.05 was considered to be significant.
